# Wnt Signaling Coordinates the Expression of Limb Patterning Genes During Axolotl Forelimb Development and Regeneration

**DOI:** 10.3389/fcell.2022.814250

**Published:** 2022-04-21

**Authors:** Alexander M. Lovely, Timothy J. Duerr, Qingchao Qiu, Santiago Galvan, S. Randal Voss, James R. Monaghan

**Affiliations:** ^1^ Department of Biology, Northeastern University, Boston, MA, United States; ^2^ Department of Neuroscience, Spinal Cord and Brain Injury Research Center, and Ambystoma Genetic Stock Center, University of Kentucky, Lexington, KY, United States; ^3^ The Peddie School, Hightstown, NJ, United States; ^4^ Institute for Chemical Imaging of Living Systems, Northeastern University, Boston, MA, United States

**Keywords:** Wnt, limb regeneration, limb development, axolotl, Fgf

## Abstract

After amputation, axolotl salamanders can regenerate their limbs, but the degree to which limb regeneration recapitulates limb development remains unclear. One limitation in answering this question is our lack of knowledge about salamander limb development. Here, we address this question by studying expression patterns of genes important for limb patterning during axolotl salamander limb development and regeneration. We focus on the Wnt signaling pathway because it regulates multiple functions during tetrapod limb development, including limb bud initiation, outgrowth, patterning, and skeletal differentiation. We use fluorescence *in situ* hybridization to show the expression of Wnt ligands, Wnt receptors, and limb patterning genes in developing and regenerating limbs. Inhibition of Wnt ligand secretion permanently blocks limb bud outgrowth when treated early in limb development. Inhibiting Wnt signaling during limb outgrowth decreases the expression of critical signaling genes, including *Fgf10*, *Fgf8,* and *Shh*, leading to the reduced outgrowth of the limb. Patterns of gene expression are similar between developing and regenerating limbs. Inhibition of Wnt signaling during regeneration impacted patterning gene expression similarly. Overall, our findings suggest that limb development and regeneration utilize Wnt signaling similarly. It also provides new insights into the interaction of Wnt signaling with other signaling pathways during salamander limb development and regeneration.

## Introduction

Tetrapod limb development utilizes highly conserved signaling pathways to regulate morphogenesis. Our understanding of this process is ascribed mainly to studies performed in mice and chickens. These studies have shown that limbs arise from the lateral plate mesoderm through interactions of retinoic acid, Tbx5, and ß-catenin/Wnt signaling to activate and maintain *Fgf10* expression and promote bud outgrowth (For Review (McQueen and Towers 2020; Royle et al., 2021)). Fgf10 induces *Wnt3a* expression in the specialized epithelial structure, the apical epithelial ridge (AER), in chicks and broad epithelial expression of *Wnt3* in mice (Kengaku et al., 1998; Kawakami et al., 2001; Barrow et al., 2003; Witte et al., 2009). ß-catenin/Wnt signaling induced by Wnt3a maintains *Fgf8* expression in the AER, which interacts with Wnt5a in the distal mesenchyme to promote distal outgrowth (Yamaguchi et al., 1999; Gao et al., 2018). It is clear that Wnt signaling is used multifunctionally during limb development and integrates with other signaling pathways.

It is unclear how similar limb development is to limb regeneration. Axolotls are an important animal for studying limb regeneration, but our incomplete understanding of axolotl limb development limits our ability to study limb regeneration. Studies have observed differences between axolotl limb development and other tetrapods, such as lacking an AER (Sturdee and Connock 1975; Tank et al., 1977; Purushothaman et al., 2019). Several researchers have shown that genes expressed in the mouse AER, including *Fgf4, Fgf8, Fgf9, Fgf17, Wnt7a,* and Fgf receptors *Fgfr1-4*, are expressed in the axolotl mesenchyme (Han et al., 2001; Christensen et al., 2002; Purushothaman et al., 2019). Bickelmann et al. showed that expression of limb patterning genes *Hoxd13*, *Hoxa11*, *Gli3*, and *Etv4* differed from chicks and mice during late limb bud stages (Bickelmann et al., 2018). Lastly, axolotls develop their digits preaxially rather than the postaxial pattern of differentiation observed in amniotes (Shubin and Alberch 1986; Fröbisch and Shubin 2011; Purushothaman et al., 2019). These studies have begun to shed light on the morphological and molecular features of the developing salamander limb, but further characterization of gene expression is needed. Transcriptomic studies have shown that developmental genes are re-expressed during limb regeneration (Monaghan et al., 2009; Campbell et al., 2011; Monaghan et al., 2012; Knapp et al., 2013; Stewart et al., 2013; Voss et al., 2015; Bryant et al., 2017) and connective tissue cells in the regenerating limb become transcriptionally similar to limb bud cells (Gerber et al., 2018). However, the similarities and differences between limb development and limb regeneration have yet to be satisfactorily explained [(Tanaka 2016; Leigh and Currie 2022) for review].

We studied the Wnt signaling pathway to address this issue because it plays a multifunctional role during limb development. Wnt ligands bind ten different frizzled receptors (Fzd) and co-receptors in nearby cells, which activate several downstream signal transduction cascades including the canonical ß-catenin dependent pathway, noncanonical Planar Cell Polar pathway (PCP), and the Wnt/Ca^+^ pathway [(Komiya and Habas 2008; Wiese et al., 2018) for review]. We also chose to investigate Wnt signaling because it is necessary for appendage regeneration in zebrafish (Wehner et al., 2014), frogs (Yokoyama et al., 2007; Lin and Slack 2008), and salamanders (Kawakami et al., 2006; Ghosh et al., 2008). To determine if Wnt signaling plays similar roles in limb development and regeneration, we investigated the expression pattern of Wnt signaling genes in the developing and regenerating axolotl limbs. We also determined how pharmacological inhibition of Wnt signaling impacts the expression of limb patterning genes during development and regeneration.

## Materials and Methods

### Animal Care and Surgical Protocol

Animals were either bred at Northeastern University or acquired from the Ambystoma Genetic Stock Center at the University of Kentucky. Animals were maintained as described in Farkas and Monaghan (2015). Embryo development stages were evaluated according to (Bordzilovskaya et al., 1989; Nye et al., 2003). Juvenile white (d/d) axolotls between 3 and 6 cm in total length were used for drug inhibitions. Images in Figures 6,7 were 8 cm in total length. Animals were anesthetized using 0.01% benzocaine, and amputations were performed through the humerus just proximal to the elbow. After amputation, the protruding bone was trimmed back to the stump.

### Drug Treatments

C59 stock solution of 10 mM in DMSO was stored at −20°C until use. Treatments were performed by diluting C59 into animal rearing water, which was changed every other day for the duration of the treatment, with new drug added during each water change.

### HCR-FISH Probe Design

To design hybridization chain reaction fluorescent *in situ* hybridization (HCR-FISH) probe sets, we developed a custom web app called probegenerator (https://probegenerator.herokuapp.com/; see https://github.com/davidfstein/probegenerator for code). Probe Generator utilizes Oligominer (Beliveau et al., 2018) to identify 25mer oligos in a provided FASTA formatted sequence that conforms to HCR hybridization conditions (Hybridization temp = 37°C, NaCl concentration = 1 M, formamide concentration = 30%). Probes are then paired with two base pair spacers according to version 3 HCR (Choi et al., 2018) and aligned to the version 60DD axolotl genome using Bowtie2 (Langmead and Salzberg 2012) to select against probes that hit multiple genomic regions. Next, probe pairs are designated as 5′ untranslated region (UTR), open reading frame, or 3′ UTR. Up to 36 probe pairs were selected for each gene of interest, first selecting probe pairs in the open reading frame, then 3′ UTR, and lastly 5′ UTR (Supplemental Table S1). Probe pools were ordered as 50 pmol/oligo lyophilized pellets from Integrated DNA Technologies or as individual oligos in plate format from Eurofins Genomics. Probe pools were resuspended in TE buffer to obtain a concentration of 1 µM or combined from plates to generate a 1 µM solution and stored at −20°C.

### HCR-FISH in Whole Mounts

The following protocol was based upon protocols provided by Molecular Instruments. Tissues were collected and fixed in 4% paraformaldehyde overnight at 4°C followed by 3 × 5 minute washes in PBST (PBS +0.1% Tween). Tissues were dehydrated with 25% MeOH/75% PBST for 10 min, followed by 50% MeOH/50% PBST, 75% MeOH/25% PBST, 100% MeOH all on ice. Tissues were then transferred to fresh 100% MeOH and stored at −20°C overnight. The next day, the MeOH series was reversed on ice up to 100% PBST, followed by another 10 min PBST wash. Blastemas were then treated with 10 μg/ml Proteinase K (NEB) in PBST for 15 min at room temperature, followed by 4% paraformaldehyde for 20 min at room temperature. Tissues were then washed 3 × 5 minutes in PBST at room temperature. Hybridization buffer was then added at 37°C for 5 min followed by a 37°C incubation with fresh hybridization buffer for 30 min. Probes were diluted 1:200 in hybridization buffer, and samples were incubated overnight at 37°C in 1.5 ml tubes. Tissues were washed in prewarmed probe wash buffer 4 × 15 min at 37°C followed by 2 × 5 min 5XSSCT washes at room temperature. The buffer was replaced with amplification buffer and incubated for 30 min at room temperature and then replaced with a 1:50 dilution of snap-cooled hairpins and incubated in the dark overnight. Samples were then washed in 5xSSCT for 5 min, then 2 × 30 min, and then 5 min at room temperature. Tissues were then mounted in 1.5% low melting temperature agarose into capillaries with a diameter just larger than the tissue (Zeiss). Once set, mounted tissues were washed for 10 min in PBS followed by incubation in EasyIndex (LifeCanvas Technologies) overnight at 4°C.

**FIGURE 1:**
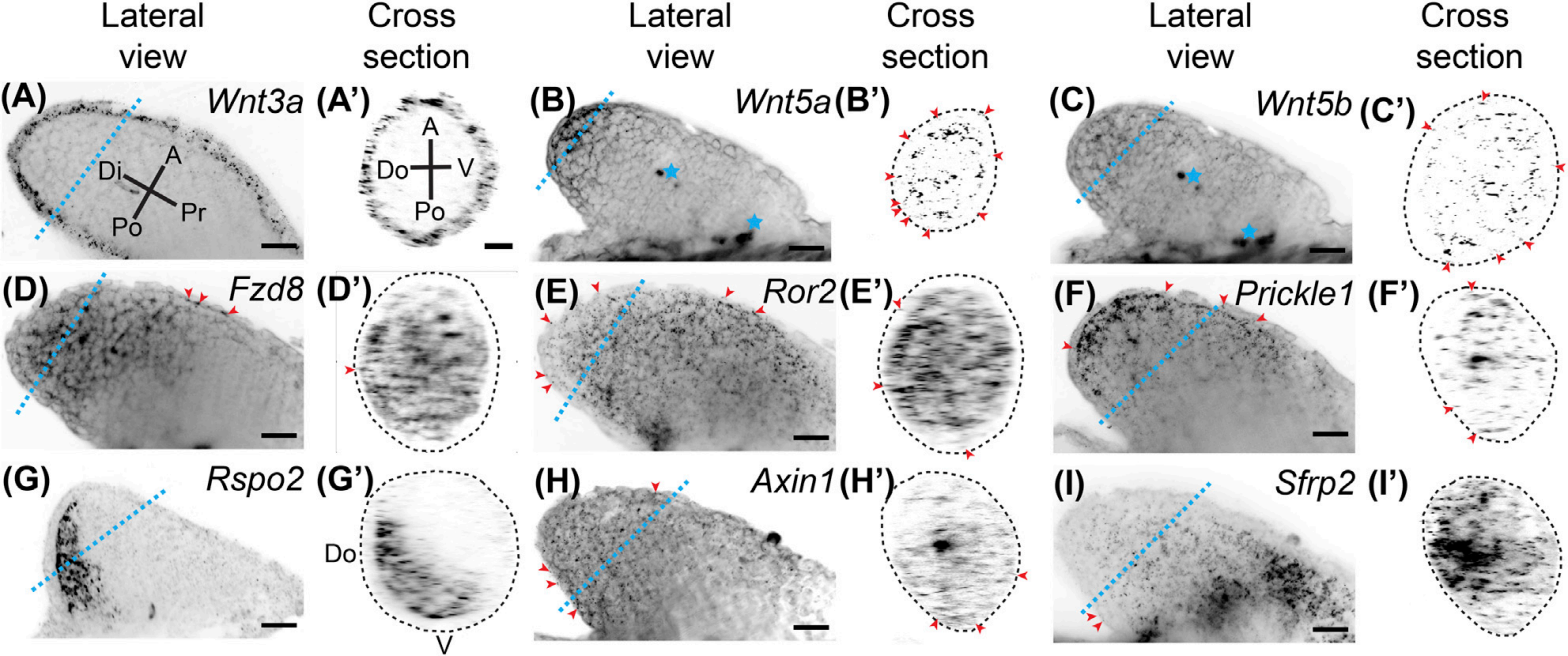
Expression of Wnt signaling genes in stage 46 developing axolotl limb buds. **(A)** Coronal view of *Wnt3a* ligand expression in a Stage 46 limb bud showing broad expression throughout the epithelium. Di is distal, A is anterior, Pr is proximal, and Po is posterior. The blue dotted line indicates the plane shown in the cross section. **(A**′**)** Cross-sectional view of the limb shown in **(A)**. A is anterior, V is ventral, Po is posterior, Do is dorsal. Notice there is no difference in expression between the anterior, posterior, dorsal, or ventral axes (APDV). **(B)** The ligand *Wnt5a* expression in the limb bud. Expression is strong in the basal layer of the distal epithelium and distal mesenchyme. Blue stars indicate regions of non-specific signal or autofluorescence of tissue, which was most often muscle in the flank of the embryo. **(B′)** Cross-sectional area of *Wnt5a* limb. Red arrowheads indicate expression of *Wnt5a* in the epithelium around the entire circumference of the limb. The dotted lines in the cross-sectional view represents the outside of the epithelium. **(C)** The ligand *Wnt5b* expression was generally weak in the developing limb bud and mainly mesenchymal. **(C′)** Sparce *Wnt5b* expression in the mesenchyme and even less in the epithelium highlighted with red arrowheads. **(D)** The Wnt receptor *Fzd8* expression in the limb bud. Broad mesenchymal expression was observed throughout the distal half of the limb bud. Red arrowheads indicate the few positive spots in the epithelium. **(D′)** Cross-sectional view of the limb in **(D)**. Notice the expression of *Fzd8* across the APDV axes. **(E)** Expression of the *Wnt5a* receptor, *Ror2*. Expression was observed across all axes throughout the entire limb bud mesenchyme with very little expression in the epithelium, highlighted with red arrowheads. **(E′)** Cross section of *Ror2* expression showing the strong expression along the APDV axes. **(F)** Expression of the downstream target of *Wnt5a*, *Prickle1*, in the limb bud. *Prickle1* mRNA dots were more concentrated distally and anteriorly. Some expression was also observed in the epithelium, highlighted with red arrowheads. **(F′)** Cross-section of limb in **(F)**. **(G)** The alternative Wnt ligand, *Rspo2*, showed completely mesenchymal expression primarily in the posterior region of the limb bud. **(G′)** The cross-sectional view shows *Rspo2* is expressed dorsally and posteriorly in the mesenchyme. **(H)** The Wnt target gene, *Axin1*, was lowly expressed throughout the limb bud in both the mesenchyme and epithelium. **(H′)** Cross-section of *Axin1* limb showing low, but broad expression. **(I)** The Wnt inhibitior, *Sfrp2*, in the limb bud showing high expression, especially in the proximal portions of the limb. **(I′)** Cross-section of *Sfrp2* expressing limb shows expression across the APDV axis, mainly in the mesenchyme. Scale bars in all panels are 50 µm.

Images were obtained on a Z.1 Light-sheet microscope with dual side illumination with a 20x plan neufluar Clr immersion objective. A single stack was selected from the image, and Denoising was performed in Zen Blue with default settings. Images were then rotated, cropped, and an inverted grey scaled lookup table was applied to each of the three image channels. Gaussian blur with a radius of 1 was performed, and brightness and contrast were adjusted using the auto function in Fiji with minor manual adjustments for image presentation. Scale bars were added at 50 µm and saved as RGB tiffs for generating the figures. Slice projections are found in Figure 1.

Wholemount samples were virtually resliced using Arivis Imaging Platform Version 3.5 to show cross-sections of limb buds. First, two 180° views were fused and the cross-sectional area of the limb bud was chosen that showed the most abundant dorsoventral and anteroposterior gene expression pattern.

Animations were generated by first generating segmentation masks of whole mount HCR-FISH by manually segmenting the limb bud using segmentation editor in ImageJ as described in Duerr et al. (2020). Masks were combined in Napari and the Napari-animation plugin was used to generate videos (Sofroniew et al., 2021).

### HCR-FISH in Tissue Sections

The following was based upon protocols provided by Molecular Instruments with some modifications in tissue collection. Fresh tissues were placed in 100% optimal cutting temperature media prechilled on ice and frozen on an aluminum block sitting in a bath of liquid nitrogen. Frozen samples were then stored at −80°C. Cryosections were taken at 10 μm, stored in the cryostat for the remainder of the tissue collection (approximately 15 min), and then fixed in 4% paraformaldehyde for 15 min at RT in lockmailer microcope slide jars. Slides were then washed 3 × 5 min in PBS and then placed in 70% EtOH at 4°C overnight to permeabilize the tissue sections. Slides were washed twice in PBS for 5 min each, followed by two 5 min washes in clearing solution (4% SDS, 200 mM Boric acid, pH 8.5), followed by two PBS washes for 5 min each. Sections were prehybridized at 37°C in hybridization buffer (Molecular Instruments) for 15 min. Probe pools (1 µM) were diluted 1:200 in 37°C hybridization buffer, and incubated on tissue sections overnight at 37°C under parafilm in a humidified chamber. Slides were then washed at 37°C in prewarmed probe wash buffer (Molecular Instruments) for 4 × 15 min. Fluorescently-labeled hairpins (Molecular Instruments) were heated to 95°C for 90 s and cooled at room temperature in the dark for 30 min before use. Amplification buffer (Molecular Instruments) was then applied on sections for 10 min at room temperature followed by a 1:50 dilution of hairpins in amplification buffer and incubated under parafilm overnight in a humidified chamber at room temperature in the dark. Sections were then washed 3 × 15 min in 5xSSCT at room temperature, stained with DAPI for 5 min, washed in PBS for 5 min, and then mounted in Prolong Gold under a 1.5# coverslip. Images were collected using a Zeiss LSM 880 confocal microscope using a 20x plan apo objective using the Airyscan fast mode. Tiles were overlaid in Zeiss Zen Software. HCR dots were identified using the RS-FISH (Bahry et al., 2021) Fiji plugin (Schindelin et al., 2012) and the same parameters were used for treated and untreated samples. Dots were overlaid on to the corresponding DAPI image for presentation purposes. Close-up images were adjusted for brightness and contrast and underwent a Gaussian Blur with a sigma of 0.5.

### Statistical Analysis

Hierarchical clustering was performed using Morpheus with metrics 1 - Pearson correlation with average linkage (http://software.broadinstitute.org/morpheus). Graphs throughout the manuscript were generated using PlotsOfData (Postma and Goedhart 2019) and organized in Adobe Illustrator. Two-tailed Student’s t-test with unequal variance was performed when comparing two groups.

## Results

### Wnt Signaling Gene Expression During Limb Development

Wnt signaling is integral to tetrapod limb development. To examine Wnt signaling gene expression during axolotl limb development, we performed whole-mount HCR-FISH on stage 46 developing axolotl limb buds (Figure 1). We found that *Wnt3a* was expressed throughout the epithelium with expression in the dorsoventral and anteroposterior axes. This expression pattern differs from AER expression in chicks (Kawakami et al., 2001; Kengaku et al., 1998), and no expression in mouse limb bud epithelium (Witte et al., 2009) (Figures 1A,A′). *Wnt5a* was highly expressed in the distal basal epithelium and mesenchyme (Figures 1B,B′), while *Wnt5b* was expressed mainly in the distal mesenchyme (Figures 1C,C′); both of these patterns are similar to expression patterns in developing mouse limbs (Martin et al., 2012; Yamaguchi et al., 1999). We observed broad *Fzd8* expression in mesenchyme with lesser expression in the most proximal regions (Figures 1D,D′). Although *Fzd8* is expressed in developing mouse limbs on embryonic day 11.5 (Summerhurst et al., 2008), chick limb buds do not express it (Nohno et al., 1999). The Wnt5a receptor, *Ror2*, which activates the PCP signaling pathway in the developing mouse limb (Gao et al., 2011), was strongly expressed throughout the limb bud mesenchyme similar to mice, but also lesser expression in the epithelium (Figures 1E,E′) (Matsuda et al., 2001). The downstream target of Wnt5a and Ror2, *Prickle1*, also showed similar expression to *Wnt5a*, although *Prickle1* was not as strongly expressed in the epithelium (Figures 1F,F′). This expression pattern is similar to chick limb development (Cooper et al., 2008), while mice also express *Prickle1* in the AER (Bekman and Henrique 2002). An alternative ß-catenin/Wnt ligand, *Rspo2*, was highly expressed posteriorly and dorsally (Figures 1G,G′), contrasting with AER expression in mice (Bell et al., 2008). Axin1, a protein that binds and is involved in degradation of ß-catenin, was very lowly but broadly expressed throughout the limb mesenchyme and epithelium (Figures 1H,H′). We investigated *Axin1* rather than the highly-expressed *Axin2* because *Axin1* is up-regulated during limb regeneration (Voss et al., 2018). The secreted Wnt signaling inhibitor, *Sfrp2*, was highly expressed in the proximal mesenchyme with less expression in distal regions (Figures 1I,I′), which deviates from mice where *Sfrp2* is expressed in the early condensing chondrocytes (Leimeister et al., 1998). Overall, ß-catenin/Wnt and PCP pathway ligands were expressed in the epithelium (*Wnt3a* and *Wnt5a*) and mesenchyme (*Wnt5a*, *Wnt5b,* and *Rspo2*), while Wnt receptors were more highly expressed in the mesenchyme (*Fzd8* and *Ror2*). Although our analysis was not exhaustive, our results show strong expression of Wnt signaling genes during limb development and significant differences between developing axolotl limbs and amniotes. These differences were observed for *Wnt3a*, *Fzd8*, *Prickle1*, *Rspo2*, and *Sfrp2*. Our data suggest that both ß-catenin/Wnt and PCP signaling pathways are active in the mesenchyme of the developing limb bud.

### Wnt Signaling is Necessary for Limb Development

**FIGURE 2:**
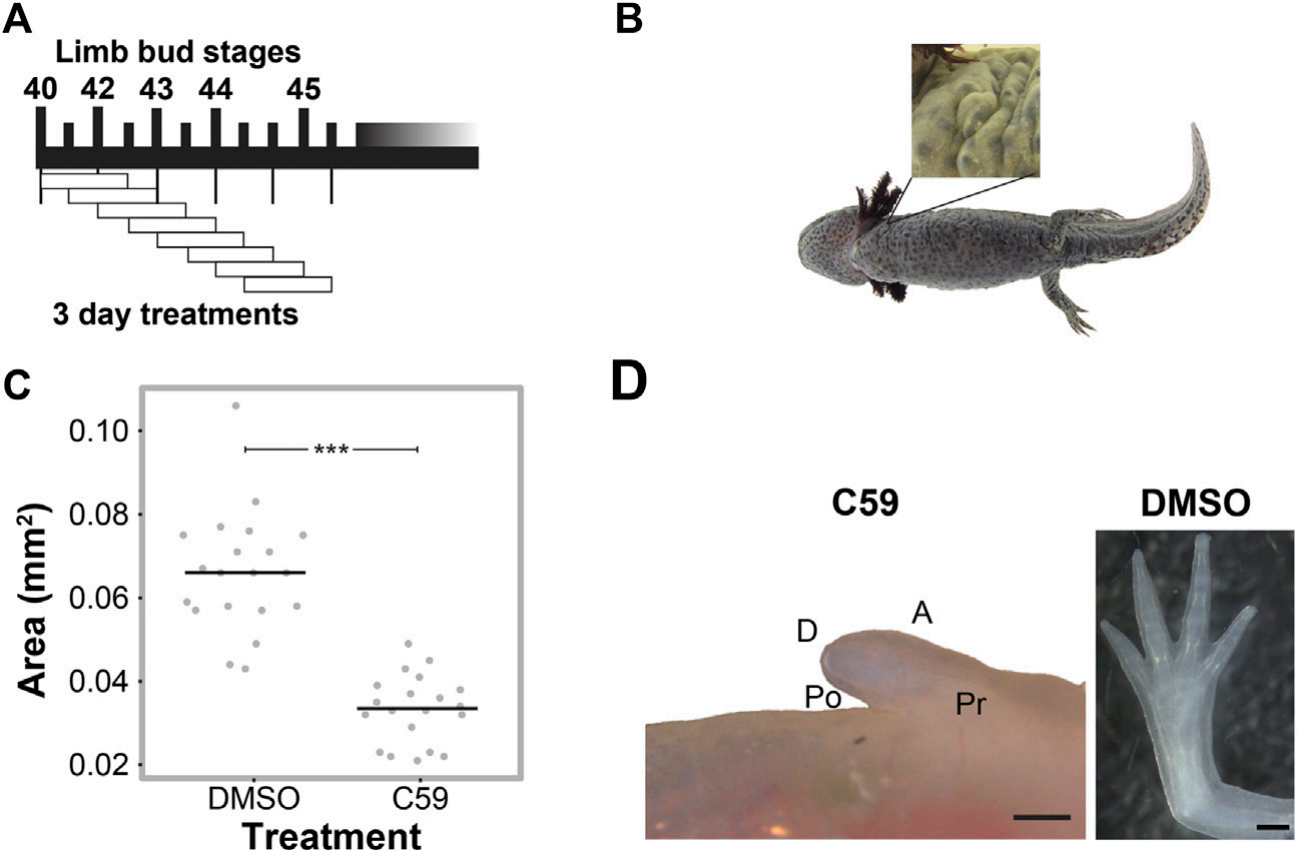
C59 treatment of developing axolotl limbs. **(A)** Treatment scheme with 10 µM C59 for 3 days intervals. Animals were allowed to grow indefinitely after treatment. **(B)** Image of a C59-treated stage 40 animal 2 years after treatment. The zoomed-in panel shows a complete lack of a limb. **(C)** Graph showing inhibition of limb bud outgrowth after 7 days of 5 μM C59 treatment starting at stage 42. Two-tailed Student’s t-test ***p < 0.0001. *n* = 20 embryos per group. **(D)** An example of a permanent limb defect after 7 days of C59 treatment starting at stage 42. Left image shows a C59-treated limb 12 weeks after treatment compared to a control limb on the right. Po is posterior, D is distal, A is anterior, and Pr is proximal. Scale bars in are 500 µM.

We next determined if Wnt secretion is required at specific time points for limb development using the well-characterized Porcupine enzyme inhibitor, C59 (Proffitt et al., 2013; Ponomareva et al., 2015), which blocks all Wnt ligand secretion. To do this, we treated animals for three-day intervals, starting at stage 40 (Figure 2A). We found permanent inhibition of limb development in all five animals treated at stage 40 (Figure 2B), which did not occur if animals were treated starting at stage 41 or beyond. These results suggest that Wnt signaling is necessary for the early stages of limb bud outgrowth and that limbs can recover from short intervals of C59 treatment after stage 40.

To determine if Wnt inhibition impacts later stages of limb development, we treated stage 42 limbs for a longer interval of 7 days and tracked their growth trajectories. Limb bud size was significantly smaller after 7 days of treatment (Figure 2C), which led to permanent truncation at the mid/distal humerus in 18 of 22 limbs (Figure 2D). Two animals had no limb buds, and two limbs developed a spike that contained segments with joint-like structures. These results suggest that the absence of Wnt ligand secretion inhibits limb outgrowth, which could have been due to a loss of cell signaling in the zone of polarizing activity (ZPA), epithelium, or mesenchyme of the developing limb.

### Wnt Signaling Regulates the Expression of Developmental Limb Patterning Genes

**FIGURE 3:**
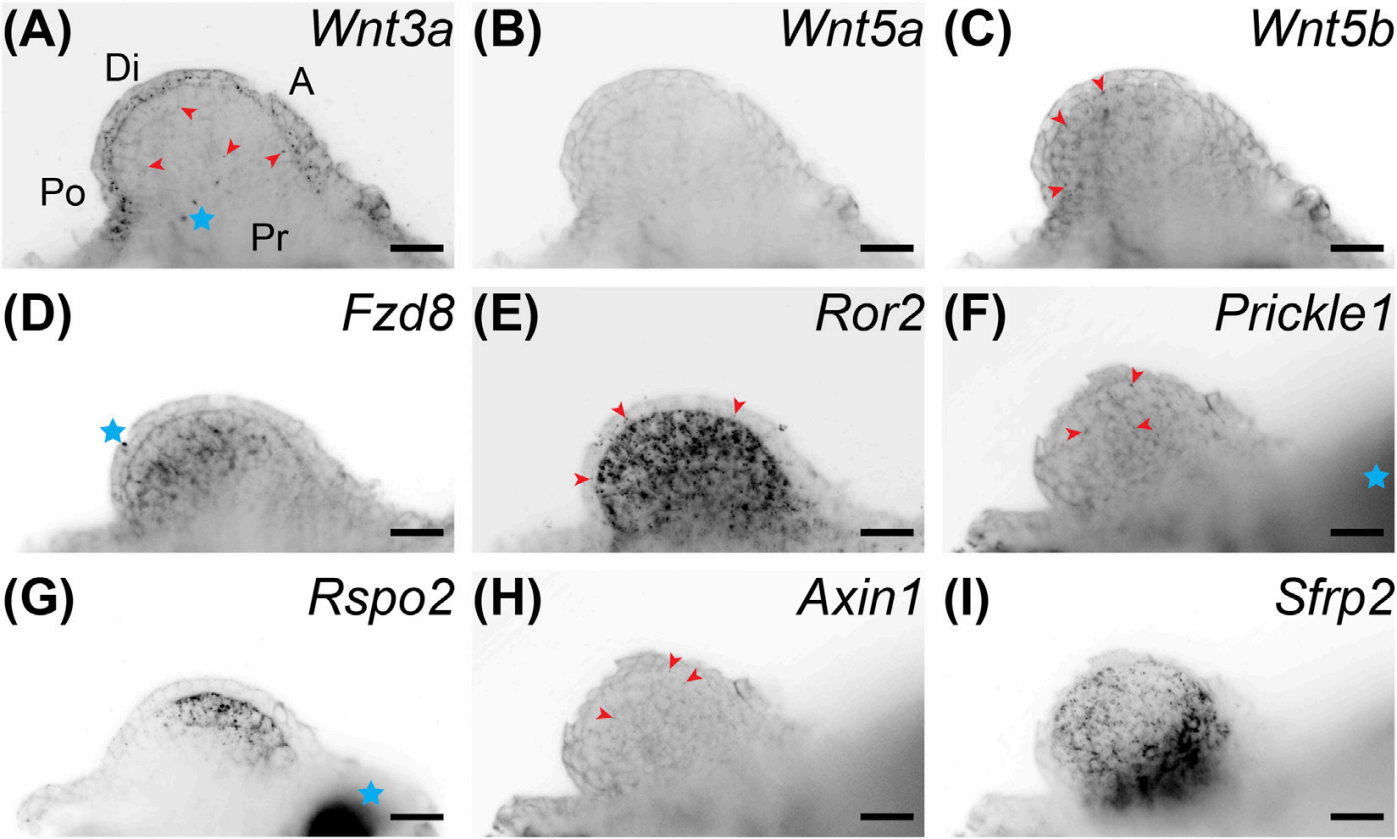
Coronal single z-plane images of whole mount HCR-FISH of Wnt signaling genes after 5 μM C59 treatment. Limbs were treated starting at stage 42 and collected on day 6 of treatment. **(A)** *Wnt3a* expression after C59 treatment. The pattern of expression is the same as untreated limbs (Figure 1A), but less pronounced. Red arrowheads show some staining in the mesenchyme although most staining is in the epithelium. The blue star indicates two non-specific dots. Po is posterior, D is distal, A is anterior, and Pr is proximal. **(B)** *Wnt5a* expression is essentially absent after C59 treatment. **(C)** Wnt5b is still lowly expressed in the same mesenchymal pattern as untreated limbs with minor staining in the epithelium (Figure 1C). **(D)** *Fzd8* shows decreased staining after treatment, but the same pattern of expression in the mesenchyme with little in the epithelium. The blue star indicates a non-specific signal on the outside the of the limb. **(E)** *Ror2* showed strong expression after C59 treatment with most expression in the mesenchyme and some in the epithelium, highlighted with red arrowheads. **(F)** Prickle1 lost most expression after C59 treatment, only retaining some expression in the mesenchyme, highlighted with red arrowheads. **(G)** *Rspo2* switched from posterior expression to an anterior mesenchymal expression domain after C59 treatment. The blue star indicates autofluorescent muscle. **(H)** Axin1 expression was minimal in limb buds treated with C59. The few positive signals are highlighted with red arrowheads. **(I)** Sfrp2 continued strong expression after C59 treatment. Scale bars in all panels are 50 µm.

We performed whole-mount HCR-FISH to determine if Wnt inhibition changed developmental gene expression patterns. We first looked at gene expression of Wnt signaling genes after C59 treatment. Although limbs were much smaller after treatment, expression continued for many Wnt genes, including *Wnt3a*, *Wnt5b*, *Fzd8*, *Ror2*, *Prickle1*, *Rspo2*, and *Sfrp2* (Figures 3A–I). In fact, drug treatment led to higher expression for the Wnt receptor *Ror2* and the Wnt inhibitor *Sfrp2* (Figures 3E,I). In contrast, *Wnt5a* and *Axin1* were nearly absent while *Rspo2* switched its expression from posterior to an anterior region (Figures 3B,G,H).

**FIGURE 4:**
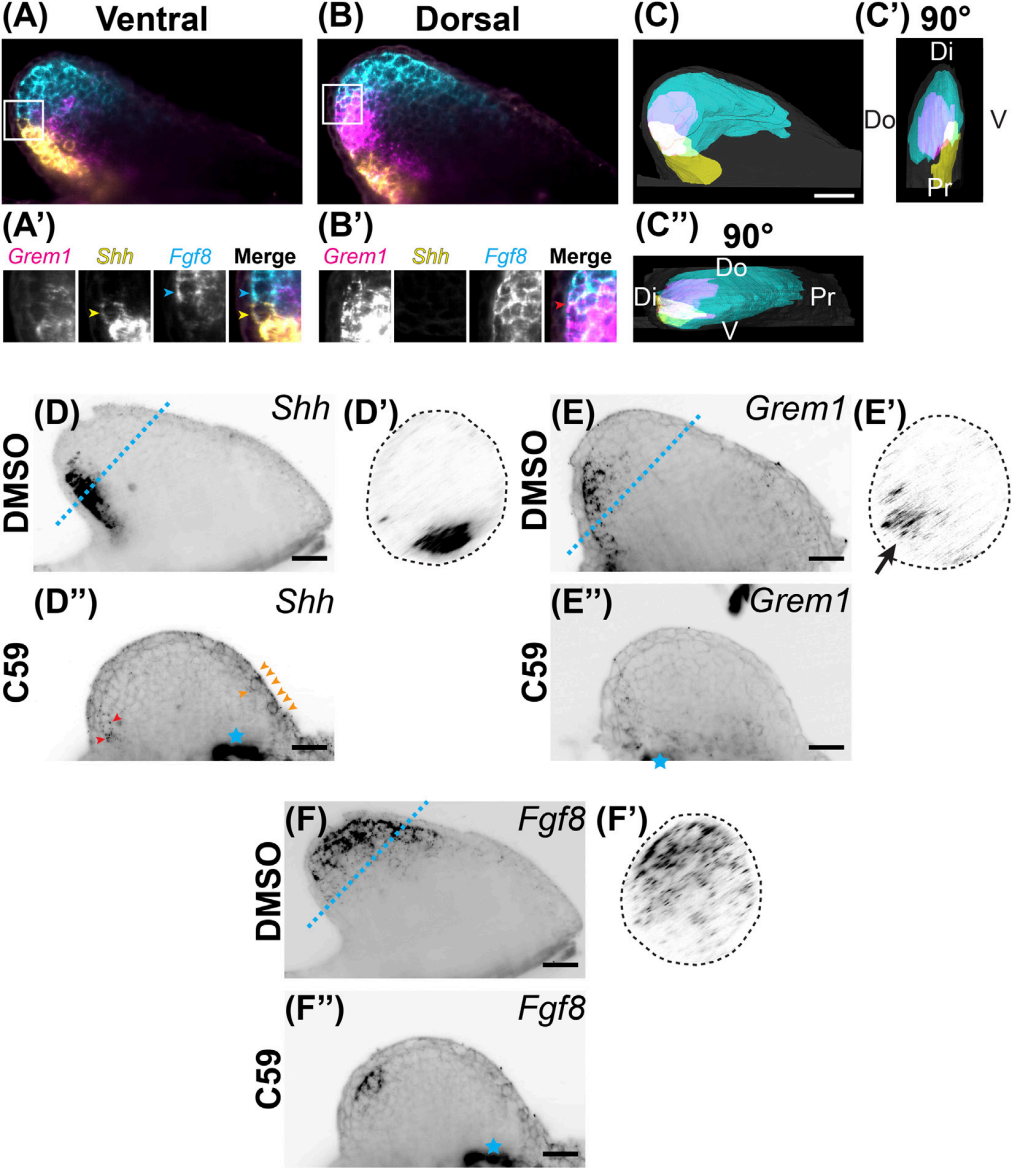
Expression of *Shh*/*Fgf8*/*Grem1* in the developing limb. **(A)** Light-sheet microscopy of HCR-FISH for *Grem1* (magenta), *Shh* (yellow), and *Fgf8* (cyan) in the ventral region. **(A′)** Zoomed-in images of the region highlighted by the white box in **(A)**. Notice the lack of overlap between the *Shh*-expressing cell highlighted with the yellow arrowhead and the *Fgf8*-expressing cell highlighted with the cyan arrowhead. **(B)** A coronal optical slice collected from the dorsal region of the limb bud. **(B′)** Images are close-ups of the boxed region in **(B)**. Notice the overlap in expression between *Grem1* and *Fgf8*. **(C)** Segmentation of *Shh*, *Fgf8*, *Grem1* expression domains of the stage 46 limb bud shown in **(A,B)**. Scale bar: 200 µm. **(C′)** A 90° rotation showing a distal/posterior view of the limb. Dorsal (Do), ventral (V), distal (Di), and proximal (Pr) regions of the limb are highlighted. **(C”)** A 90° rotation showing a distal/anterior view of the limb. Do, V, Di, and Pr are highlighted. **(D)** Single z-plane image of Shh expression in the stage 46 limb bud. **(D′)** The cross sectional view of the blue dotted line in D. Notice the posterior/ventral expression domain. **(D”)** *Shh* expression after C59 treatment. Red arrowheads show the decrease of *Shh* expression in the posterior domain. Orange arrowheads indicate a new anterior expression domain in the mesenchyme and epithelium. **(E)** *Grem1* expression in an untreated limb bud showing mesenchymal posterior expression. **(E′)** Cross sectional view of the blue dotted line in **(E)**, showing the posterior/dorsal expression domain of *Grem1*. **(E”)** Grem1 expression after C59 treatment showing a strong decline in gene expression in the posterior mesenchyme. **(F)** Fgf8 expression in the distal anterior mesenchyme of the limb bud. **(F′)** Cross section of the blue dotted line in **(F)** showing the anterior mesenchymal expression of *Fgf8* with a slight skew towards the dorsal portion of the limb bud. **(F”)** *Fgf8* expression after C59 treatment shows a decrease in the expression domain except in the most posterior distal portion of the limb. The blue star indicates autofluorescence of the flank muscle.

We next examined if Wnt inhibition impacted the Shh, Grem1, Fgf8 signaling loop known to be active in vertebrate limbs (Zúñiga et al., 1999). We observed that *Fgf8* was expressed in the distal mesenchyme with more broad expression in the anterodorsal region. *Shh* had strong expression in the posterior mesenchyme, with the region trending towards the ventral region and a sharp boundary between the *Shh* and *Fgf8* domains (Figures 4A–C). *Grem1*, the Bmp antagonist that relays Shh and Fgf signaling (Sun et al., 2000; Zúñiga et al., 1999), was expressed mainly between the *Shh* and *Fgf8* domains with higher expression dorsally and some overlap with *Shh* and *Fgf8* (Figures 4A–C; Supplementary Video S1). Our observation of overlap between *Shh* and *Grem1* is also observed in Xenopus limb buds (Wang et al., 2015), but not chicks (Scherz et al., 2004). Wnt inhibition led to a significant decrease in all three transcripts with a shift of *Fgf8* more posteriorly and a small anterior ectopic expression domain of Shh in the mesenchyme and epithelium (Figures 4D–F).

**FIGURE 5:**
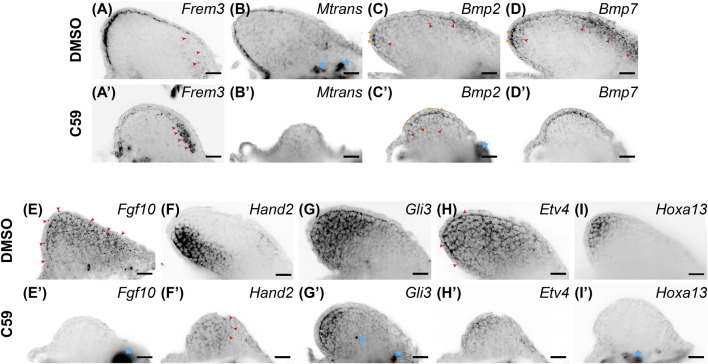
Gene expression of limb patterning genes in stage 46 developing axolotl limb buds. **(A)** Expression of *Frem3* in the distal epithelium. Some mesenchymal expression can be observed in the anterior/proximal mesenchyme highlighted with red arrowheads. **(A′)** Epithelial expression of *Frem3* decreased and the anterior mesenchymal expression was significantly increased after C59 treatment shown by red arrowheads. **(B)** Epithelial expression of Mtrans with a scewing towards the posterior side of the limb. **(B′)** C59 treatment greatly decreased expression of Mtrans in the epithelium. **(C)** *Bmp2* expression is expressed in the basal epithelium are indicated with orange arrowheads and mesenchymal expression is highlighted with red arrowheads. **(C′)** *Bmp2* expression had the same expression domains after C59 treatment, highlighted by orange arrowheads (epithelium) and red arrowheads (mesenchyme). The blue star indicates autofluorescence. **(D)** *Bmp7* expression in the epithelium indicated with orange arrowheads and mesenchymal expression in the distal and anterior mesenchyme indicated with red arrowheads. **(D′)** *Bmp7* expression continued after C59 treatment with a decrease in the anterior mesenchyme. **(E)** *Fgf10* expression throughout the mesenchyme and lower levels in the epithelium. **(E′)** *Fgf10* expression was completely absent after C59 treatment. **(F)** *Hand2* expression in the posterior limb mesenchyme. **(F′)** *Hand2* expression decreased and expanded in anterior regions of the mesenchyme, highlighted by red arrowheads. **(G)** *Gli3* expression mainly in the mesenchyme. **(G′)** Decreased *Gli3* staining in the mesenchyme. Blue stars indicate non-specific staining. **(H)** Broad mesenchymal staining of *Etv4* throughout the limb bud with a few positive spots in the epithelium, highlighted by red arrowheads. **(H′)** Decreased *Etv4* expression after C59 treatment, but with the same expression pattern as **(H)**. **(I)** Distal mesenchymal expression of *Hoxa13*. **(I′)** Lack of *Hoxa13* expression after C59 treatment. The blue star indicates autofluorscent muscle. Scale bars in all panels are 50 µm.

Considering that a lack of Wnt signaling inhibits AER formation in chicks and mice, we expected C59 to impact epithelial gene expression. Indeed, Wnt inhibition decreased the expression of most epithelial genes. *Frem3*, known to be expressed in the mouse limb bud epithelium (Chiotaki et al., 2007), switched expression from the mainly posterior limb epidermis to the anterior mesenchyme after C59 treatment (Figures 5A,A′). *Mtrans*, a transcript previously shown to have high expression in the regenerating limb wound epidermis (AMEX60DD102055433.1) (Campbell et al., 2011; Monaghan et al., 2012), was completely abrogated in the developing limb epithelium with C59 treatment (Figures 5B,B′). In addition, the C59 treatment decreased epithelial expression of *Wnt3a* (Figures 1A, 3A) and *Wnt5a* (Figures 1B, 3B), while *Bmp2* and *Bmp7* expression in the epithelium and mesenchyme were only mildly impacted (Figures 5C,D′).

Mesenchymal gene expression was also significantly impacted after Wnt inhibition. The primarily mesenchymal expression of *Fgf10* was completely absent after C59 treatment (Figures 5E,E′). *Hand2*, a posteriorly expressed transcription factor necessary for Shh expression in mice (Galli et al., 2010), had its posterior mesenchymal expression pattern decreased and expanded throughout the anteroposterior mesenchyme (Figures 5F,F′). The mesenchymal expression of *Gli3* (Figures 5G,G′), and mainly mesenchymal *Etv4* (Figures 5H,H′) decreased expression, while the autopod identity gene, *Hoxa13*, was completely absent in C59-treated limbs (Figures 5I,I′). These results show that inhibition of Wnt signaling decreases the overall expression of patterning genes in both the epithelium and mesenchyme while causing several patterning genes to adjust their expression domains, especially along the anteroposterior axis.

### Wnt Signaling is Necessary for Limb Regeneration

**FIGURE 6:**
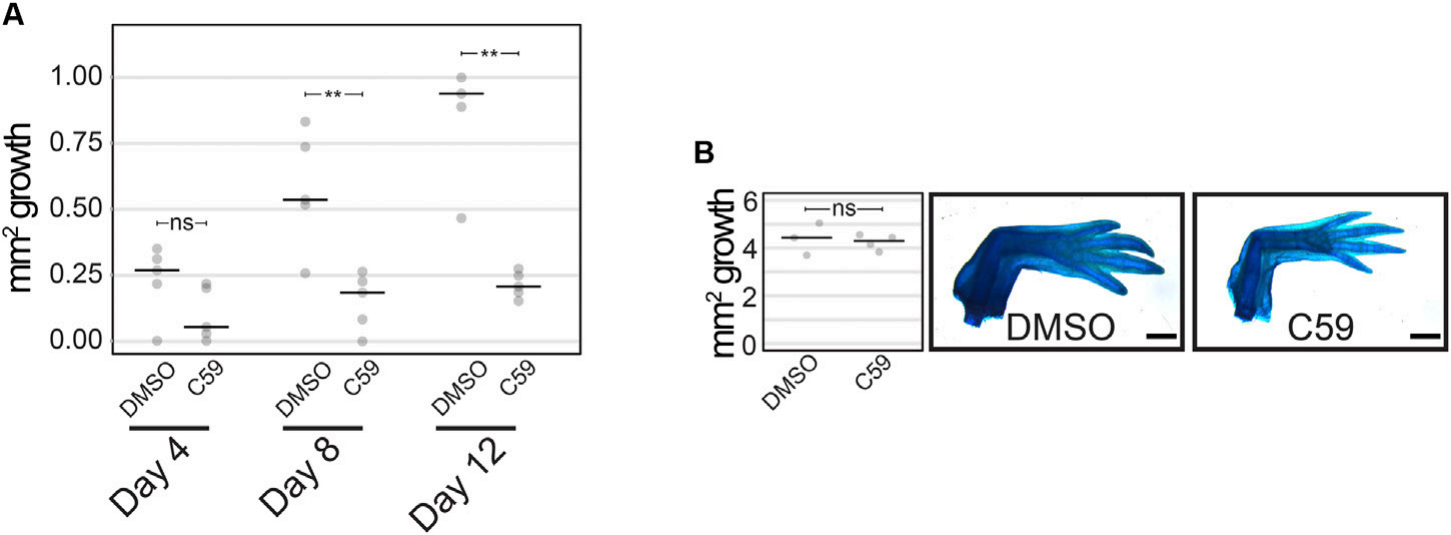
Limb regeneration after Wnt inhibition. **(A)** Quantification of limb growth after C59 treatment starting at 3 dpa. Treated limbs were smaller than control limbs at 8 dpa (*p* = 0.0092; Student’s t-test) and 12 dpa (*p* = 0.0031; Student’s t-test). **(B)** Limb regeneration of untreated (DMSO) and treated (C59) limbs 1 month after removal from C59 (12 dpa).

We next determined if Wnt signaling was necessary for limb regeneration and its possible mechanism of action. Treatment with 5 µM C59 from 3–12 dpa (*n* = 5) decreased the area of regenerated tissue, with the first significant difference detected at 8 dpa (*p* = 0.0092; Student’s t-test) and a lack of growth in C59-treated limbs after that point (Figure 6A). These data suggest that active Wnt ligand secretion is necessary for blastemal growth. Once animals were removed from C59 treatment at 12 dpa, regeneration growth recovered, and limbs fully regenerated to control levels by 1 month later (Figure 6B; *p* = 0.719; Student’s t-test). These results suggest that the pool of blastema cells necessary for limb regeneration are not lost due to Wnt inhibition, and the cells are still competent to recommence regeneration once Wnt ligands become available. A similar scenario occurs when axolotl limbs are denervated, recommencing regeneration once nerves reinnervate the limb.

### Wnt Signaling Regulates the Expression of Limb Patterning Genes During Regeneration

**FIGURE 7:**
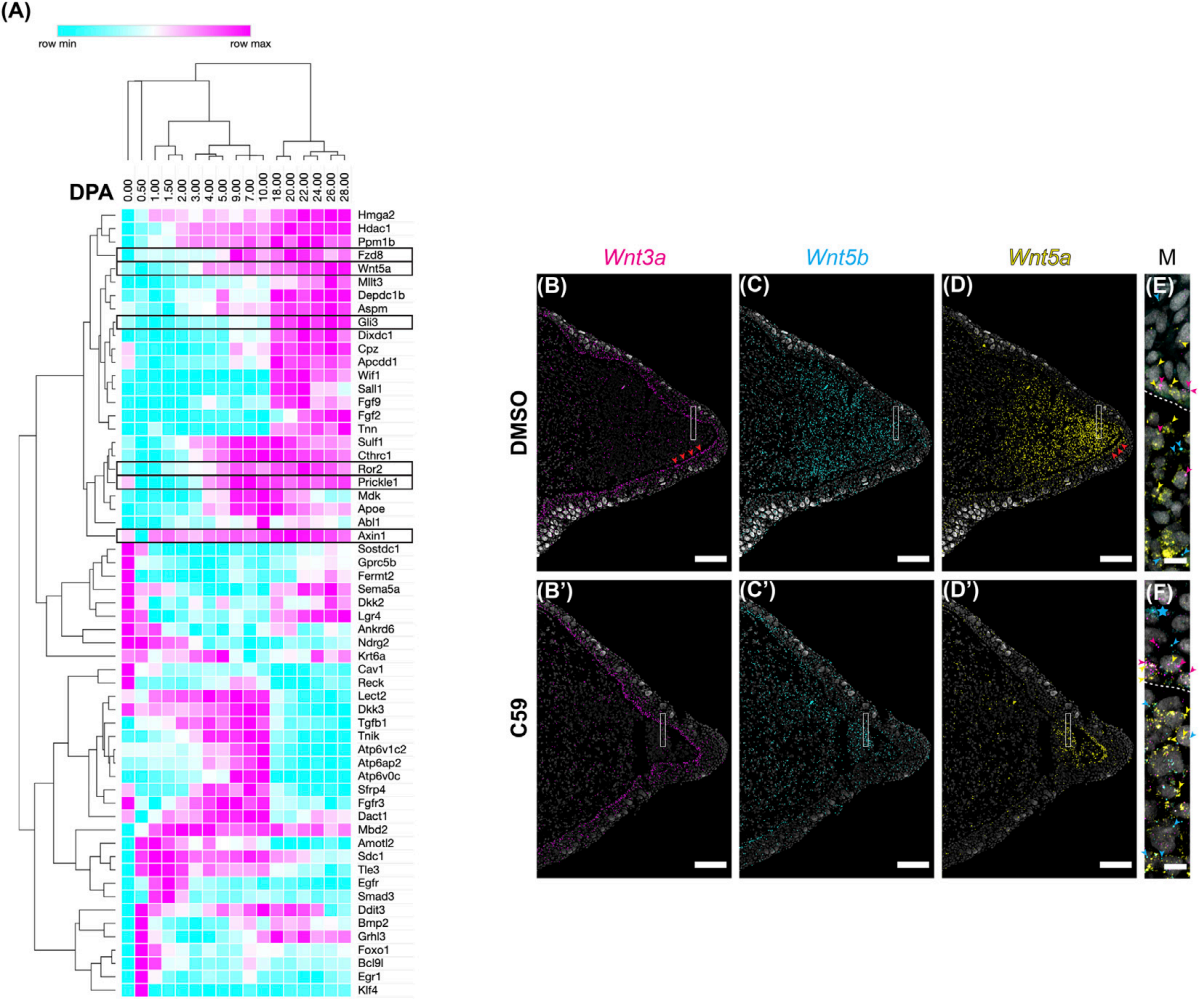
Wnt ligand expression during limb regeneration. **(A)** Hierarchical clustering of 59 Wnt signaling genes during 28 days of limb regeneration. Days post amputation are presented on the x axis. Genes are presented on the y axis. Genes evaluated by HCR-FISH are highlighted with boxes. **(B–D)** HCR-FISH was performed on a coronal section of a forelimb blastema at the mid bud stage, 20 dpa. C59 treatment started on 18 dpa. The few non-specific dots located outside of the limb were removed for clarity. Scale bars **(B–D′)** are 250 µm. **(B)** *Wnt3a* expression in the mid blastema showing strong expression in the basal epidermis, highlighted by red arrowheads. Some mesenchymal expression is present in the mesenchyme (highlighted with magenta arrowheads in the merged image, **(E)**. **(B′)** *Wnt3a* was minimally impacted from 48 h of C59 treatment. **(C)** Broad mesenchymal expression of *Wnt5b*. Few spots were observed in the epithelium. **(C′)** Decreased *Wnt5b* expression in C59 treated limb with same expression pattern as **(C)**. **(D)** *Wnt5a* expression showing strong mesenchymal and basal epithelium expression higher near the distal blastema. Red arrowheads highlight expression in the basal epithelium. **(D′)** *Wnt5a* expression retained only in the most distal mesenchyme and basal epithelium after C59 treatment. **(E,F)** Close-up images were adjusted for brightness and contrast and Gaussian blurred with a radius of 1. Dotted lines indicate the epithelial boundary. Scale bars in **(E,F)** are 20 µm. **(E)** Close-up merged image of boxed area in **(B–D)** showing *Wnt3a*, *Wnt5b*, and *Wnt5a* expression. Magenta arrowheads highlight *Wnt3a* expression, cyan arrowheads highlight *Wnt5b* expression, and yellow arrowheads highlight *Wnt5a* expression in a DMSO-treated limb. **(F)** Close-up merged image of boxed area in **(B′–D′)** showing *Wnt3a*, *Wnt5b*, and *Wnt5a* in C59 treated limb. Blue star indicates an autofluorescent cell in the epithelium.

Next, we investigated the expression of Wnt signaling genes during forelimb regeneration. We first mined a public transcriptomic dataset that characterized gene expression by Affymetrix microarray after upper arm amputation over the first 28 days post-amputation (dpa) with ten biological replicates at each time point (Voss et al., 2018). We selected 447 unique genes with the gene ontology term associated with Wnt signaling (GO term GO:0198738 “cell signaling by wnt”) that were also on the Affymetrix microarray (*n* = 274). Genes were chosen for analysis only if they changed at any time point (*p* < 0.05 using ANOVA statistical test = 186 genes) and had at least a two-fold change from uninjured limbs (*n* = 59; Supplementary Table S2). Hierarchical clustering of these genes showed dynamic expression patterns over time, including upregulation of Wnt signaling genes at blastema formation, approximately 4 dpa (Figure 7A). Based upon these results, Wnt signaling is likely active during limb regeneration.

We next used multiplexed HCR-FISH in tissue sections to determine the expression patterns of Wnt ligands with and without C59 treatment. We studied the mid-stage blastema because it closely resembles the stage 46 developing limb. Overall, patterns were very similar to limb development with *Wnt3a* primarily expressed in the basal epidermis and still expressed after C59 treatment (Figures 7B,B′). *Wnt5b* was primarily expressed in the blastema mesenchyme and marginally declined with C59 treatment (Figures 7C,C′). In contrast, *Wnt5a* significantly decreased expression in the basal epidermis and blastema mesenchyme, mimicking the response observed in limb development (Figures 1B, 3B). Early blastema (6 dpa) and early/mid blastema (11 dpa) stages showed the same expression patterns except that *Wnt5b* was absent at the early blastema stage (Supplementary Figure S1).

**FIGURE 8:**
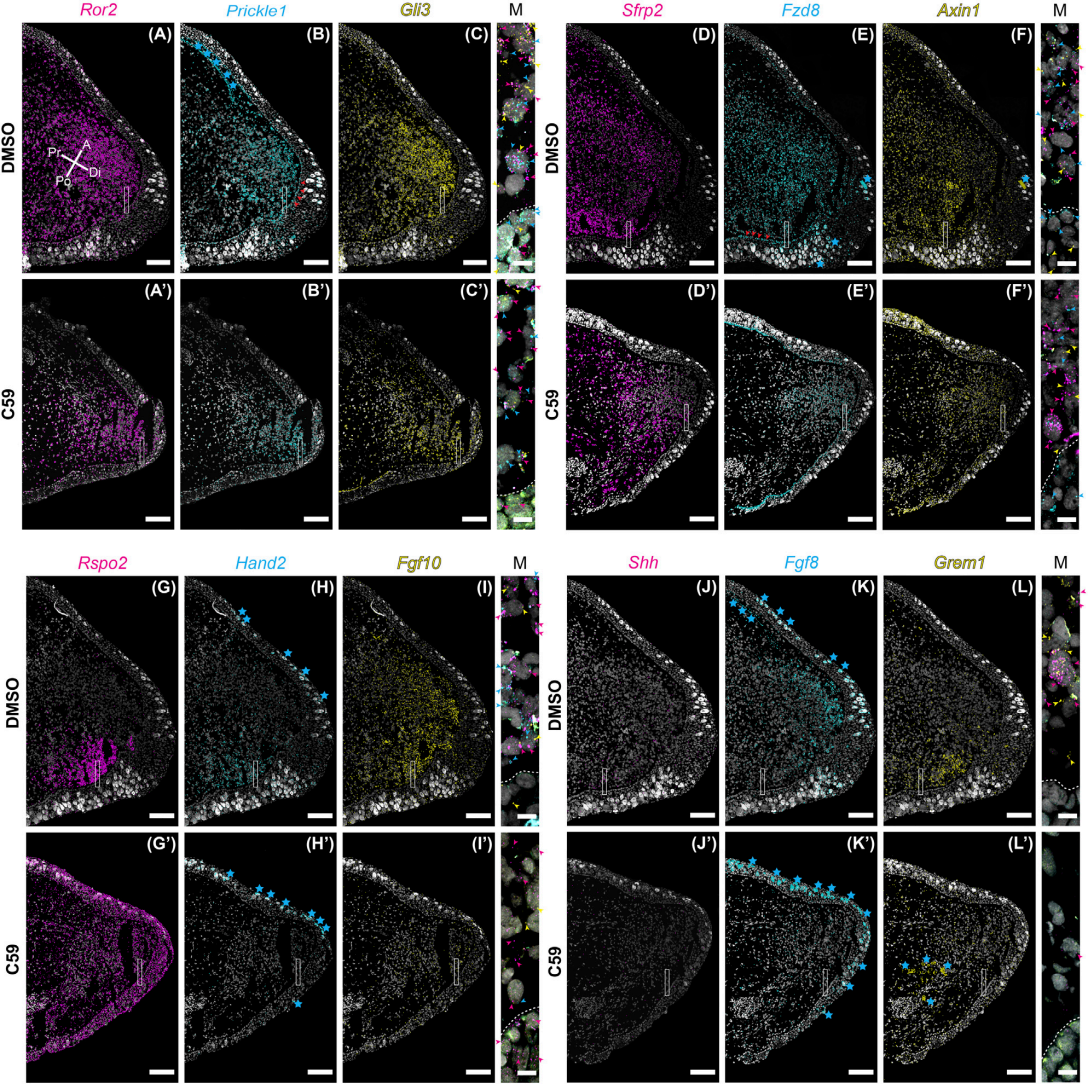
Gene expression of limb patterning genes in mid bud axolotl forelimb blastemas. **(A–L′)** HCR-FISH was performed on coronal sections of a forelimb blastema at the mid bud stage, 20 dpa. C59 treatment started on 18 dpa. The few non-specific dots located outside of the limb were removed for clarity. Scale bars **(A–L′)** are 250 µm. Scale bars in close-up merged images were 20 µm. **(A)** *Ror2* expression in a DMSO-treated limb showing very strong expression throughout the mesenchyme and lesser expression in the epithelium. Pr is proximal, A is anterior, Di is distal, and Po is posterior. **(A′)** Generally decreased expression of *Ror2* in a C59-treated limb. **(B)** *Prickle1* expression in the blastema mesenchyme and basal epithelium, indicated by red arrowheads. Blue stars indicate autofluorescent basal lamina in the epidermis and the dermis. **(B′)** Expression of *Prickle1* after C59 treatment showing similar, but less frequent signal. **(C)** Strong *Gli3* expression in the mesenchyme of the blastema. **(C′)** Expression of *Gli3* after C59 treatment showing similar, but less frequent signal. The merged image of **(A–C)** shows variable expression across cells for *Ror2*, *Prickle1*, and *Gli3*, highlighted by colored arrowheads. **(D)** *Sfrp2* strongly expressed throughout the blastema mesenchyme. **(D′)** *Sfrp2* expression similar in a C59-treated limb compared to the DMSO-treated limb. **(E)** Mesenchymal and basal epithelial expression of *Fzd8*. Red arrowheads highlight expression in the basal epithelium. Blue stars indicate autofluorescent Leydig cells in the epithelium. **(E′)** Similar expression in the C59-treated limb, but at lower levels. **(F)** Low expression of *Axin1* throughout the blastema mesenchyme with very low expression in the epithelium. **(F′)** Similar *Axin1* expression pattern in a C59-treated limb compared to control limbs, except at a lower level. **(G)** *Rspo2* expression in the posterior mesenchyme of the blastema. **(G′)** *Rspo2* broadly expressed in both the mesenchyme and epithelium of a C59-treated blastema. **(H)** Posterior mesenchymal expression of *Hand2* in the limb blastema. Autofluorescent Leydig cells are highlighted with blue stars. **(H′)** Lack of expression for *Hand2* in the C59-treated limb blastema. **(I)** *Fgf10* mainly expressed in the mesenchyme. **(I′)** *Fgf10* expression nearly absent in the C59-treated blastema. **(J)** Posterior expression of *Shh* in few, but highly expressing cells in the posterior blastema mesenchyme. The lack of stronger expression is likely due to the tissue section not beingincluding much of the posterior *Shh* domain. Strong *Shh* expression is observed in whole-mount staining in Supplementary Figure S2. **(J′)** Very little *Shh* expression was observed in C59-treated limbs, which was confirmed by whole-mount imaging. **(K)** Distal/anterior mesenchymal expression of *Fgf8* in the regenerating blastema. Blue stars indicate autofluorescent Leydig cells and dermis. **(K′)** *Fgf8* transcripts absent in the C59-treated limb. **(L)** Posterior mesenchymal expression of *Grem1* in a broader domain than *Shh*. **(L′)** Very little *Grem1* expression in the C59-treated limb.

Considering C59 treatment had a significant impact on blastema growth, we wondered if C59’s impact might be due to a lack in the expression of genes associated with distal outgrowth. To test this, we performed multiplexed HCR-FISH of mid-stage blastemas for the genes impacted during development with and without C59 treatment. Overall, gene expression patterns during regeneration were similar to developmental gene expression patterns (Figures 8A–L). A minor difference we observed was that *Ror2* (Figure 8A), *Prickle1* (Figure 8B), *Fzd8* (Figure 8E), and *Axin1* (Figure 8F) were more abundant in the blastema epithelium compared to development.

The response to C59 was also very similar to developing limbs. Some genes had a minor qualitative decrease in expression, including *Ror2* (Figures 8A,A′), *Prickle1* (Figures 8B,B′), *Gli3* (Figures 8C,C′), *Sfrp2* (Figures 8D,D′), *Fzd8* (Figures 8E,E′), and *Axin1* (Figures 8F,F′), but were not substantially different from control limbs. The most striking difference between limb development and regeneration was that C59 induced broad expression of *Rspo2* in both the mesenchyme and epithelium during regeneration (Figures 8G,G′) compared to a posterior to anterior switch in development (Figures 1G, 3G). Another difference was that *Hand2* was nearly absent after C59 treatment in the blastema compared to decreased expression and expansion into the anterior limb bud during development (Figures 8H,H′). We observed that *Hand2* (Figures 8H,H′), *Fgf10* (Figures 8I,I′), *Shh* (Figures 8J,J′), *Fgf8* (Figures 8K,K′), and *Grem1* (Figures 8L,L′) were all nearly absent after 48 h of C59 treatment. This observation is supported by whole-mount imaging, showing that C59 treated mid-stage blastemas had a complete lack of *Fgf10*, *Shh*, and *Fgf8* expression (Supplementary Figure S2). This observation further supports that Fgf/Shh crosstalk is important in driving distal outgrowth of the regenerating limb (Nacu et al., 2016) and that *Fgf10* is associated with the growth stages of the blastema (Christensen et al., 2002; Nacu et al., 2016). Altogether, our data suggest that limb regeneration has highly similar gene expression patterns to limb development, and inhibiting Wnt signaling has a similar impact on patterning gene expression. Based upon the complete lack of gene expression after C59 treatment of *Fgf10*, and the *Shh*, *Grem1*, *Fgf8* signaling loop, it is possible that these are direct downstream targets of Wnt signaling.

## Discussion

Salamanders have an uncommon ability to regenerate amputated limbs. Data suggests connective tissue cells near the limb amputation plane change their transcriptional profile to a state similar to cells in the developing limb bud (Gerber et al., 2018; Lin et al., 2021). It is unclear if this similarity at the cellular level also occurs at the level of tissue patterning. Therefore, it is necessary to comprehensively study the similarities and differences between limb development and regeneration. The current understanding is incomplete, partly due to the lack of studies of salamander limb development using molecular markers (Han et al., 2001; Christensen et al., 2002; Satoh et al., 2007; Ghosh et al., 2008; Monaghan and Maden 2012; Shimokawa et al., 2013; Bickelmann et al., 2018; Purushothaman et al., 2019). Here, we addressed this problem by studying the expression of genes involved in cell and patterning during limb development and regeneration, emphasizing Wnt signaling.

Overall, we observed inhibition of limb outgrowth after perturbing Wnt ligand secretion during both limb development and regeneration. Based upon the large body of research on tetrapod limb development, we can devise several likely scenarios to explain the phenotypes we observed in our study. Based upon previous work (Hill et al., 2006; Kawakami et al., 2001), inhibition of *Wnt2b* secretion may have directly impacted the expression of *Fgf10* during limb development (Kawakami et al., 2001; Ng et al., 2002)*,* which would then decrease *Wnt3a* expression (Kengaku et al., 1998; Kengaku et al., 1997), leading to decreased *Fgf8* and *Shh* (Kengaku et al., 1998). In both limb development and regeneration, our data suggest a direct connection between Wnt and *Fgf10*, as we observed a complete loss of *Fgf10* expression after C59 treatment (Figures 5E,E′, 8I,I′). *Fgf8* and *Shh* both decreased expression and shifted posteriorly during development, and were nearly absent after C59 treatment (Figure 4), suggesting that *Fgf8* and *Shh* may be direct or secondary consequences of Wnt’s regulation. Alternatively, C59 could also have inhibited Wnt3a secretion in the epithelium, decreasing *Fgf8* and *Shh* (Kengaku et al., 1998). Regardless, loss of *Fgf* expression would, in turn, stop the Shh/Grem1/Fgf feedback loop, decreasing the Bmp inhibitor *Grem1*, which would then increase Bmp signaling precociously and prevent distal outgrowth in developing and regenerating limbs. In support of this observation in development, we observed continued expression of *Bmp2* and *Bmp7* after C59 treatment. Future experiments will determine if the epithelial expression of *Wnt3a* is driving loss of the Shh/Grem1/Fgf signaling loop and an increase in Bmp signaling. Lastly, inhibition of Wnt5a and Wnt5b secretion could decrease outgrowth and prevent distal limb differentiation (Yamaguchi et al., 1999; Gao et al., 2011; Gao et al., 2018). While it is clear that several scenarios could explain our results, we present new insights about the hierarchy of signaling during axolotl limb development, and we made progress in determining unique and conserved aspects of salamander limb outgrowth with other tetrapods.

We also observed mesenchymal gene expression for some genes typically expressed in the epithelium of other tetrapods. The most striking contrasts were the mesenchymal expression of *Fgf8* (Han et al., 2001; Christensen et al., 2002; Wang and Beck 2014; Purushothaman et al., 2019; Schloissnig et al., 2021), and *Rspo2* (Bell et al., 2008). Mesenchymal *Fgf8* and *Rspo2* correlate with the lack of a functional AER in the salamander limb. Still, it is unclear whether the lack of *Fgf8* or *Rspo2* expression in the epithelium causes the absence of an AER. Others have observed mesenchymal expression of *Wnt7a* in salamanders, which is expressed in the dorsal epithelium of amniotes (Shimokawa et al., 2013). Overall, it is likely that several genes typically expressed in the AER of the developing amniote limb have mesenchyme expression domains in axolotls. It is not yet determined the consequence of this shift on signaling centers in the developing limb. Furthermore, it will be interesting to see whether this pattern of mesenchymal gene expression is present in other salamanders such as newts.

A distinct phenotype we observed after C59 treatment during limb development was the absence of an autopod (Figure 2D). This phenotype could be partially explained by the complete lack of expression for the autopod-specific gene, *Hoxa13*, suggesting that the autopod is not specified in C59-treated limbs (Figures 5I,I′). In mice, ablation of *Wnt5a* leads to reduced cell proliferation and a lack of distal elements (Yamaguchi et al., 1999). Alternatively, epidermal *Wnt3a* expression in chicks (Kengaku et al., 1998), and *Wnt3* in mice (Barrow et al., 2003; Soshnikova et al., 2003), are required for AER formation and maintenance leading to defects in the autopod. Together, these scenarios could decrease the expression of distal identity genes and truncate the developing limb. In addition to the lack of distal outgrowth, we observed a substantial shift in anteroposterior gene expression. In particular, *Hand2*, *Rspo2*, and *Frem3* expression domains shifted anteriorly, in contrast to the loss of anterior *Gli3* expression and posterior shift of *Shh*, *Grem1*, and *Fgf8*. Overall, these severe phenotypes suggest dysfunctional anterior-posterior patterning after Wnt inhibition.

To determine similarities of limb development with regeneration, we also studied Wnt gene expression during regeneration. Wnt signaling’s role in salamander limb regeneration was first demonstrated by adenovirus overexpression of the intracellular ß-catenin/Wnt pathway inhibitor, *Axin1,* which generated a spike rather than a patterned regenerate. This study also overexpressed the secreted inhibitor *Dkk*, blocking limb regeneration (Kawakami et al., 2006). In support of these findings, overexpression of *Wnt5a* by vaccinia virus, which inhibits ß-catenin/Wnt signaling, also blocked axolotl limb regeneration (Ghosh et al., 2008). The Wnt inhibitor IWR-1-endo, which increases ß-catenin destruction, also inhibited newt limb regeneration (Singh et al., 2012). Overactivation of Wnt signaling is also detrimental to axolotl limb regeneration, possibly through decreased innervation and defects in skeletal differentiation (Wischin et al., 2017). Together, these studies provide strong evidence the proper regulation of Wnt signaling is necessary for salamander limb regeneration. Our study builds upon these observations by showing the expression patterns of genes involved in ß-catenin/Wnt and PCP signaling and provides further evidence for the importance of Wnt signaling. Our data also suggest that Wnt signaling is upstream of *Fgf10*, and the *Fgf8*, *Shh*, *Grem1* loop signaling. However, Shh antagonism can be rescued with ß-catenin/Wnt agonists in newts, suggesting that Shh is upstream of ß-catenin/Wnt (Singh et al., 2012). Further work is needed to elucidate whether ß-catenin/Wnt or PCP are the direct upstream regulators of the Fgf/Shh feedback loop or whether their decrease is due to an indirect effect on blastemal growth.

Recent data suggest that the ‘typical’ amniote postaxial mode of limb development is likely a derived condition, and the salamander preaxial mode is ancestral. This data raises the possibility that regenerative ability may depend upon the re-deployment of an ancestral mode of limb development that is retained in salamanders but lost in amniotes, emphasizing the importance of understanding salamander limb development at the gene expression level (Trofka et al., 2021). Here we aimed to meet this goal using a well-known pharmacological inhibitor of Wnt ligand secretion and gene expression analysis to determine the functions of Wnt signaling during axolotl limb development and regeneration. We show that inhibited Wnt signaling influences several downstream targets, leading to defects in limb bud outgrowth and a temporary inhibition of limb regeneration. Further work will be needed to determine the specific roles of each Wnt ligand and ß-catenin/Wnt versus PCP signal transduction pathway. In future research, it will be interesting to determine the spatiotemporal dynamics of ß-catenin versus PCP signaling in developing and regenerating limbs.

## Data Availability

The original contributions presented in the study are included in the article/Supplementary Material. Raw images can be found at the Northeastern Digital Repository (http://hdl.handle.net/2047/D20389398).

## Supplementary Material

Supplementary Figure 1 HCR-FISH on coronal sections of forelimb blastemas. **(A)** Early (6 dpa) blastema showing expression of *Wnt3a*, *Wnt5a*, *Axin1*, *Sfrp2*, *Prickle1*, *Ror2*, and *Fzd8*. **(B)** Wnt ligands *Wnt5a*, *Wnt5b*, and *Wnt5a* in an 11 dpa blastema. Although expression was lower and autofluorescence was higher due to blood and the wound environment, patterns were similar to mid/late blastema stages shown in Figures 7, 8. Gaussian blur was performed with a radius of 2 on all images and brightness and contrast modified for presentation purposes.

Supplementary Figure 2
**(A)** Light-sheet imaging of a mid-bud blastema for *Fgf8* (yellow), *Fgf10* (magenta), and *Shh* (cyan). **(A′)** An optical slice through the image shown in **(A)**. **(B)** Light-sheet imaging of a limb treated with C59 for 8 days showing a lack of expression for Fgf8 (yellow), Fgf10 (magenta), and Shh (cyan). **(B′)** An optical slice through the image shown in **(B)**.

Supplementary Table 1 Probe sequences used for HCR-FISH. B1-B4 correspond to the HCR initiators used for signal amplification.

Supplementary Table 2 Genes involved in Wnt signaling that significantly changed from baseline levels at least 2 fold.
